# Substance P induces localization of MIF/α1-inhibitor-3 complexes to umbrella cells via paracellular transit through the urothelium in the rat bladder

**DOI:** 10.1186/1471-2490-6-24

**Published:** 2006-09-18

**Authors:** Pedro L Vera, Katherine L Meyer-Siegler

**Affiliations:** 1 The Bay Pines VA Healthcare System, Research & Development, Bay Pines, FL, USA; 2 University of South Florida, Department of Surgery, Tampa, FL, USA

## Abstract

**Background:**

Macrophage migration inhibitory factor (MIF) is released into the intraluminal fluid during bladder inflammation in the rat complexed to α1-inhibitor-3 (A1-I3; a rodent proteinase inhibitor in the α-macroglobulin family). The location of A1-I3 in the bladder had not been investigated. Therefore, we examined the location of A1-I3 and MIF/A1-I3 complexes in the bladder and changes due to experimental inflammation.

**Methods:**

Anesthetized male rats had bladders removed with no treatment (intact) or were injected with Substance P (SP; s.c.; saline vehicle). After one hour intraluminal fluid was removed, bladder was excised and MIF and A1-I3 levels were determined using ELISA and/or western-blotting. MIF co-immunoprecipitation determined MIF/A1-I3 complexes in the bladder. Bladder sections were immunostained for A1-I3 and MIF/A1-I3.

**Results:**

A1-I3 immunostaining was observed in interstitial spaces throughout the bladder (including submucosa) but not urothelium in intact and saline-treated rats. RT-PCR showed that the bladder does not synthesize A1-I3, therefore, A1-I3 in the interstitial space of the bladder must be plasma derived. In SP-treated rats, A1-I3 in the bladder increased and A1-I3 was observed traversing through the urothelium. Umbrella cells that do not show MIF and/or A1-I3 immunostaining in intact or saline-treated rats, showed co-localization of MIF and A1-I3 after SP-treatment. Western blotting demonstrated that in the bladder MIF formed non-covalent interactions and also binds covalently to A1-I3 to form high molecular weight MIF/A1-I3 complexes (170, 130 and 75-kDa, respectively, verified by co-immunoprecipitation). SP-induced inflammation selectively reduced 170-kDa MIF/A1-I3 in the bladder while increasing 170 and 130-kDa MIF/A1-I3 in the intraluminal fluid.

**Conclusion:**

A1-I3 and MIF/A1-I3 complexes are resident in bladder interstitium. During SP-induced inflammation, MIF/A1-I3 complexes are released from the bladder into the lumen. Binding of MIF/A1-I3 complexes to urothelial cells during inflammation suggests these complexes participate in the inflammatory reaction through activation of receptors for MIF and/or for A1-I3.

## Background

We have been studying the role of the pleiotropic and pluripotent cytokine macrophage migration inhibitory factor (MIF) in pelvic viscera inflammation. MIF, initially recognized as released by T-cells to stop macrophage migration, is expressed by different cells and involved in various functions (e.g. cell proliferation, tumorigenesis and inflammation) [1,2]. We first reported that MIF is constitutively expressed in human and rat urothelium [3,4]. Furthermore, cystitis (e.g. experimental in rats [5-7] or bacterial in humans [8]) increased levels of MIF in the intraluminal fluid (or urine) and upregulated MIF production in the bladder. Because MIF stands upstream of other inflammatory markers in the bladder [6], we showed that sequestering intraluminal MIF with MIF antisera decreased inflammatory bladder changes [9]. Thus, our experimental evidence supports the hypothesis that MIF plays a central role in the development and/or maintenance of bladder inflammation, in agreement with MIF's importance in inflammatory conditions [1].

Our recent findings of high-molecular weight MIF-complexes released into the intraluminal fluid of rats [10] and human urine [8] were unexpected because in vitro studies described a monomeric (12-kDa) and occasionally dimeric (24-kDa) MIF conformation [11], while x-ray crystallography revealed a trimeric (36-kDa) structure for MIF [12]. However, we observed that intraluminal MIF formed complexes by: (1) non-covalent associations (broken apart by lithium dodecyl sulfate and heat to yield 12-kDa MIF) and (2) covalent associations (interrupted by dithiolthreitol reducing agent [8,10]). Using mass spectroscopy, Western-blotting (WB) and co-immunoprecipitation, we demonstrated covalently associated MIF with α1-Inhibitor-3 (A1-I3) in the rat, while MIF was complexed to ceruloplasmin, uromodulin, albumin and α2-macroglobulin in human urine [8].

A1-I3 is a rodent monomeric proteinase inhibitor (apparent molecular weight 180 kDa) in the α2-macroglobulin family, with which it shares approximately 60% homology [13]. Both A1-I3 and α2-macroglobulin are synthesized by the liver, released into the circulation due to inflammation and taken up by inflamed organs [14]. A1-I3 (and other members of α2-macroglobulin family of protease inhibitors) bind to proteases at a bait region and trap them in conformational changes that prevent further action of the proteases. Once activated by protease binding to the bait-region, a receptor region is exposed that allows clearing of the protease by binding to the low-density lipoprotein (LDL) receptor and also a cytokine binding site is exposed [15-17]. Several cytokines bind to members of the α2-macroglobulin family (at sites other than the bait-region) and may participate in distributing those cytokines to specific targets [15].

We recently described MIF/A1-I3 complexes in the intraluminal fluid of the rat [10], however, the location of A1-I3 in the bladder and whether MIF/A1-I3 complexes are resident in the bladder or are the result of leakage of plasma proteins during inflammation (plasma extravasation) had not been investigated. In the present study, we examine the location of A1-I3 and MIF/A1-I3 complexes in the bladder and describe changes induced by experimental inflammation. In particular, we show that MIF/A1-I3 complexes are resident in naïve bladders and during Substance P-induced inflammation, there is translocation of MIF/A1-I3 complexes to superficial urothelial cells only along with release of MIF/A1-I3 complexes into the bladder lumen. This represents a novel finding and suggests that MIF/A1-I3 complexes (and possibly other cytokines associated with members of the α-macroglobulin family) may participate in inflammatory processes in the bladder through association with apical urothelial cells.

## Methods

All experiments were approved by the institutional Animal Care Committee and conformed to the National Institutes of Health guidelines for animal experimentation.

### Substance P-induced bladder inflammation

SP-induced bladder inflammation was described in detail previously [6,9,10,18]. Briefly, fifteen male rats (300–350 grams; Harlan; IN), anesthetized with sodium pentobarbital (60 mg/kg; i.p.) were divided into:

(1) ***Intact: ***Bladder was excised (N = 5)

(2) ***Saline: ***Bladder was isolated from the kidneys by tying the ureters distally, cutting them centrally, and draining to gauze pads. Urine was removed from the bladder and replaced with saline (0.3 ml). Rats received saline (0.3 ml; s.c.) and after 1 hour the intraluminal fluid (ILF) and bladder were removed (N = 5);

(3) ***Substance P: ***Bladder was isolated as in (2) and urine replaced with saline, but this group received substance P (SP; 40 ug/kg; s.c.) and after 1 hour the ILF and bladder were removed (N = 5).

Urine, ILF and bladders were processed for either immunohistochemistry, protein and RNA as described [5-7,9,10]. All animals were overdosed (sodium pentobarbital ;100 mg/kg; i.p.) at the end of the experiment.

### Immunohistochemistry

Frozen bladder sections (10 microns) were exposed to A1-I3 antisera (rabbit polyclonal; 1:1000; a gift from Dr. Harry van Goor); and visualized using an ABC procedure (Vector, Burlingame, California) and counterstained with hematoxylin. Double-immunofluorescence was performed using antisera to MIF (Torrey Pines Biolabs, Houston, Texas; 1:200) and A1-I3 (1:1000). Primary antisera were visualized using secondary antisera with FITC or TRITC. Control experiments included omission of the primary antisera or omission of one of the two secondary antisera (in the case of dual immunofluorescence).

### Western-Blotting and MIF ELISA procedures

MIF Western-blotting (WB) was performed under native, non-reducing and reducing conditions (Tris-glycine or NuPAGE Bis-Tris gels, Invitrogen, Carlsbad, CA) as described previously [10]. Briefly, total bladder homogenates were prepared using 10 mM CHAPS buffer (3-[(3-cholamidopropyl) dimethylammonio]-1-propane-sulfonate, 2 mM ethylenediaminetetraacetic acid and 4 mM iodoacetate in phosphate buffered saline (PBS), pH 7.2 with the addition of general protease inhibitors (Sigma, St. Louis, MO) and total protein concentrations in cleared lysates determined so that equal protein concentrations (10 μg) were loaded on all gels. For native conditions (i.e non-reducing, non-denaturing), proteins were separated on Tris-glycine gels using the reagents and as supplied by the manufacturer (Invitrogen). Under non-reducing conditions (i.e. non-reducing, denaturing), proteins were heated to 70°C in the presence of lithium dodecyl sulfate (LDS) prior to loading onto NuPAGE Bis-Tris gels (4–12%, Invitrogen). Under reducing conditions (i.e. reducing, denaturing), proteins were heated to 100°C in the presence of LDS and 50 mM dithiolthreitol prior to loading onto NuPAGE Bis-Tris gels (4–12%, Invitrogen). In addition, reducing conditions included addition of anti-oxidant (Invitrogen) to running and transfer buffer to prevent oxidation of reduced cysteine, methionine and tryptophan residues. After electrophoresis, separated proteins were transferred to PVDF. MIF protein bands were detected using biotinylated polyclonal antibody (BAF289, R&D Systems, Minneapolis, MN), strepavidin-HRP conjugates and chemiluminescent substrate (Pierce, Rockford, IL). Blots were stripped (200 mM glycine pH 2.2, 1% Tween-20, 0.1% SDS) then exposed to A1-I3 antisera (1:1000) and detected as above. Recombinant rat MIF (15 ng; Torrey Pines) was run as a standard to estimate MIF protein concentrations. Band intensities were quantified using Kodak Image Station (Kodak, Rochester, NY).

MIF concentrations in bladder homogenates and ILF were also determined using a rat MIF ELISA (Chemicon, Temecula, CA). Data are expressed as mean ng/mg of protein (bladder) and ng/ml (ILF) ± S.E.M. from each treatment group.

### Co-Immunoprecipitation procedures

Bladder homogenates (250 μg protein) were used to isolate MIF interacting proteins by co-immunoprecipitation using MIF antibody (5 μg; AB-289-PB, R&D Systems) and protein G agarose beads following the manufacturer's protocol (Kirkegaard & Perry Laboratories, Gaithersburg, MD) as described [18]. Briefly, bladder homogenate proteins that non-specifically bind to protein G were removed by a pre-clearing step in which the sample was incubated with protein G beads for 1 h at 4°C. Following the pre-clear step protein G agarose beads were discarded, anti-MIF antibody added and samples incubated overnight at 4°C with constant agitation. Freshly equilibrated protein G beads were then added and the samples incubated at 4°C for 2 h. The protein G beads were then extensively wash and bound proteins eluted by the addition of 2 × NuPAGE LDS sample buffer and heating to 95°C for 15 min. The protein G eluted proteins were removed from the beads by centrifugation, DTT added and the samples heated to 100°C. The immunoprecipitated proteins were separated by reducing NuPAGE Bis-Tris gels, transferred to PVDF and A1-I3 immunostaining bands detected as described for WB. Controls included incubation of pre-cleared samples with non-specific IgG (Sigma) and samples processed without the addition of antibody in the immunoprecipitation reaction.

### PCR procedures

Bladder and liver total RNA was isolated using TriZol reagent (Invitrogen) and reverse transcribed as described previously [7]. 2 μl aliquots of the resulting cDNA were amplified for MIF and A1-I3 using low stringency conditions: 35 cycles of 94°C 1 min, 50°C 1 min, 72°C 2 min. MIF PCR primer design was described previously [5]; MIF upstream primer: 5' CTCTCCGAGCTCACCCAGCAG 3', downstream primer: 5' CGCGTTCATGTCGTAATAGTT 3'. A1-I3 primers were designed to amplify 231 bp fragment between nucleotides 3223–3453 of mRNA (upstream primer: 5'CTCAAGTCTTTTGCCCAAGC3'; downstream primer: 5'GGAGACAACGGGATCTGTGT3'). This region spans exons 27–29 of the 58,910 bp A1-I3 gene located on chromosome 4 (NW_047696, GeneBank). PCR generated fragments were separated and detected using 2% agarose gels (E-Gel, Invitrogen). Controls for RT-PCR reactions included: 1) separate amplification of GAPDH (primers obtained from Ambion, Austin, TX) as a positive control for total RNA concentrations and reverse transcription reactions, 2) A1-I3 PCR amplification of liver cDNA as a positive control for A1-I3 expression and 3) total RNA without reverse transcriptase in PCR reaction as a negative control.

### Statistical Analyses

ELISA data were analyzed using t-tests. WB data were analyzed using t-tests (in the case of total intensity) or using Two-way ANOVA (treatment × bands) followed by post-hoc tests (Newman-Keuls) if significance was reached (Prism4; GraphPad Software, San Diego, CA). Data are presented as mean ± SEM. Significance was defined as p < 0.05.

## Results

### SP-induced co-localization of MIF/A1-I3 in umbrella cells

**Figure 1:**
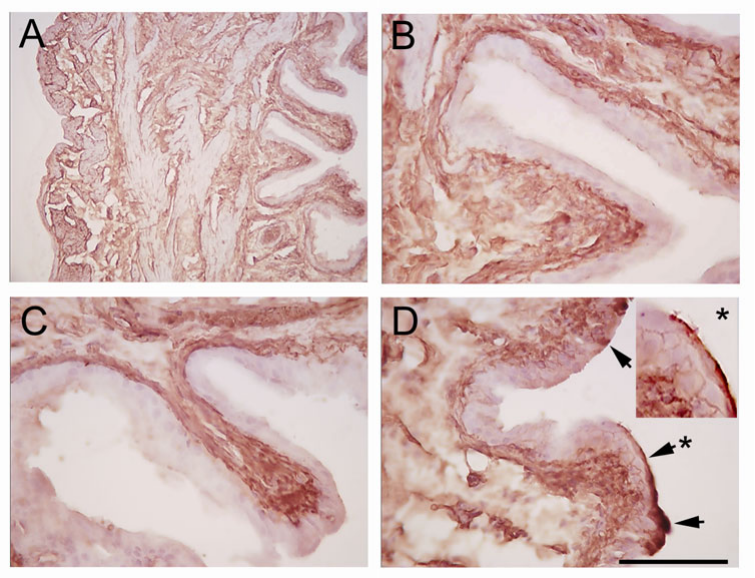
**Substance P-induced A1-I3 immunostaining in umbrella cells. **A1-I3 immunostaining in bladders from intact (A; B) and saline treated rats (C) showed A1-I3 in the interstitial spaces and in the lamina propria immediately below the urothelium, but not in the urothelium. In substance P-treated animals (D), however, A1-I3 immunostaining was detected in the urothelium, particularly on umbrella cells. Note also immunostaining surrounding (but not intracellular) in the basal and intermediate layers of the urothelium (see insert) in SP-treated rats. Calibration bar: A = 400 um; B, C, D = 100 um; insert = 50 um.

A1-I3 immunostaining was demonstrated in the intact bladder submucosa and interstitial space (Fig 1A;B). However, A1-I3 immunostaining was absent from the urothelium. Similar findings were observed for saline treated animals and Substance P treated animals (Figure 1C;1D). Substance P treatment resulted in A1-I3 immunostaining of the urothelium that previously showed no A1-I3. The staining appeared to traverse the urothelium (Fig 1D and insert), and surround urothelial cells (Fig. 1D and insert), but did not appear to be cytoplasmic, in the basal and intermediate layers of the urothelium. Superficial cells, however, showed intense cytoplasmic A1-I3 immunostaining (Fig 1D; and insert).

**Figure 2:**
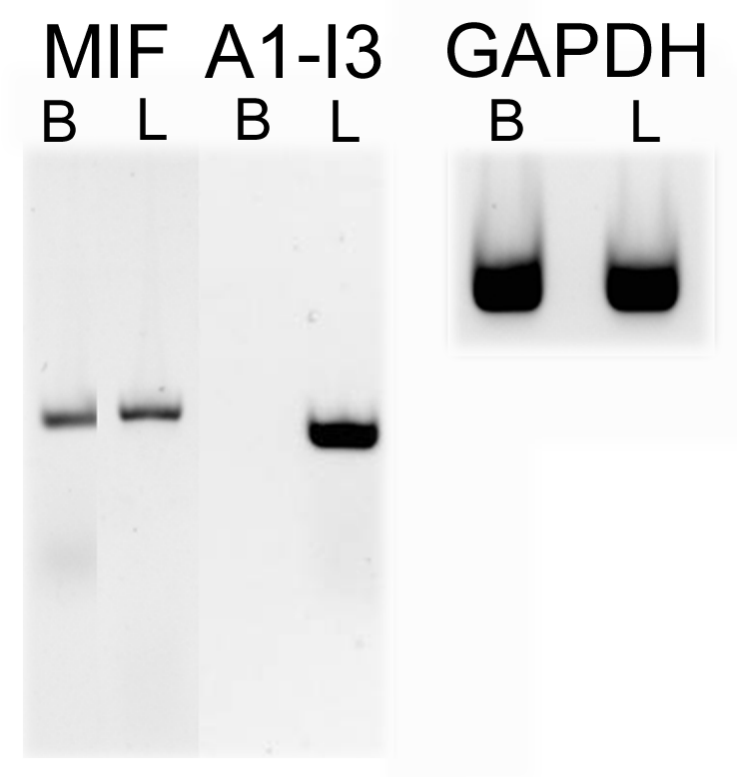
**MIF and A1-I3 expression in the bladder and the liver. **MIF and A1-I3 mRNA were amplified by RT-PCR. Resulting PCR products were resolved on 2% agarose gels. A single MIF band was detected in both the bladder (B) and liver (L). A1-I3 message was detected only in the liver (L), but not in the bladder (B). GAPDH RT-PCR documented similar amounts of total RNA were extracted and amplified in both bladder (B) and liver (L).

RT-PCR analysis confirmed our previous results that documented MIF expression in the rat bladder and also demonstrated MIF expression in the rat liver (Fig. 2). Under low stringency RT-PCR conditions, no A1-I3 expression was observed in the bladder (Fig. 2). Therefore since the mRNA for this protein was not found in the bladder, it is likely that A1-I3 is synthesized some place else (most likely, liver) and then transported into the bladder and stored in the tissues.

**Figure 3:**
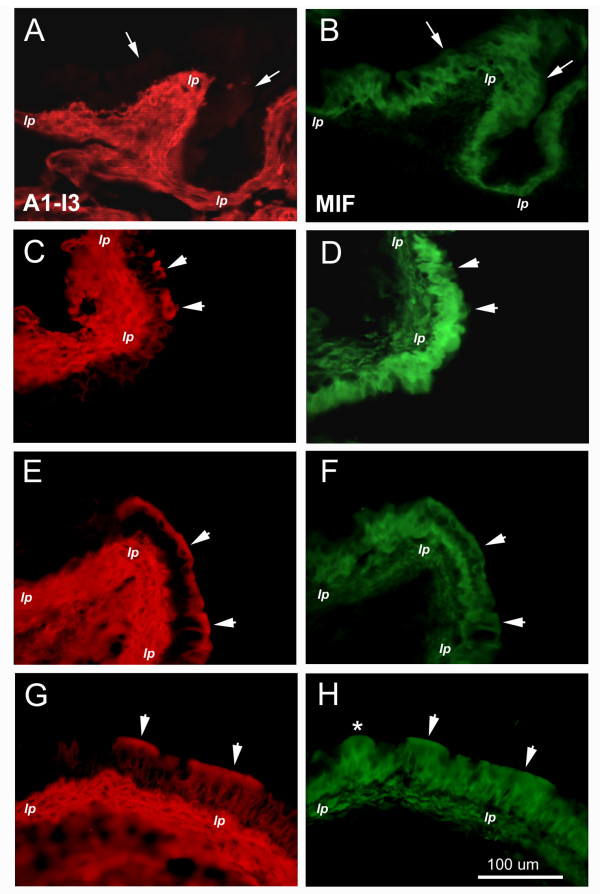
**Colocalization of MIF/A1-I3 in umbrella cells after SP-treatment. **Dual-immunofluorescence for A1-I3 and MIF in bladder sections from saline (A, B) and SP-treated rats (C–H). Bladders from saline-treated rats showed no A1-I3 in the urothelium (A; edge of unstained urothelium marked by long arrows) while the lamina propria (lp) showed significant A1-I3 immunostaining. On the other hand, MIF is present in the urothelium of saline-treated rats (B), mainly in basal and intermediate cells. Representative sections from three (3) different animals show that after substance P treatment, A1-I3 immunostaining is seen in umbrella cells (C, E, G; arrows) that also co-localized MIF (D, F, H; arrows). In addition, increased staining in the lamina propria (lp) can be observed (D, F, H). Asterisk (H) shows area of superficial urothelium with MIF immunostaining but lacking A1-I3 immunostaining (G). Calibration bar = 100 um.

Using dual immunofluorescence, strong A1-I3 immunostaining was observed in the lamina propria, immediately below the urothelium (Fig 3A) of saline treated rats, but no A1-I3 was observed in the urothelium. Strong MIF staining was observed in the urothelium while weak MIF immunostaining was observed in the lamina propria (Fig 3B), in agreement with our previous reports [3]. In SP-treated rats, A1-I3 immunostaining was now detected in the urothelium, particularly umbrella cells that also showed weak to moderate MIF immunostaining (Fig 3C–H). Although A1-I3 immunostaining surrounding urothelial cells was observed throughout parts of the urothelium, the staining in the umbrella cells appeared cytoplasmic. In addition, increased MIF immunostaining was observed in the lamina propria (Fig 3C–H).

### SP-induced changes in bladder MIF/A1-I3 complexes

**Figure 4:**
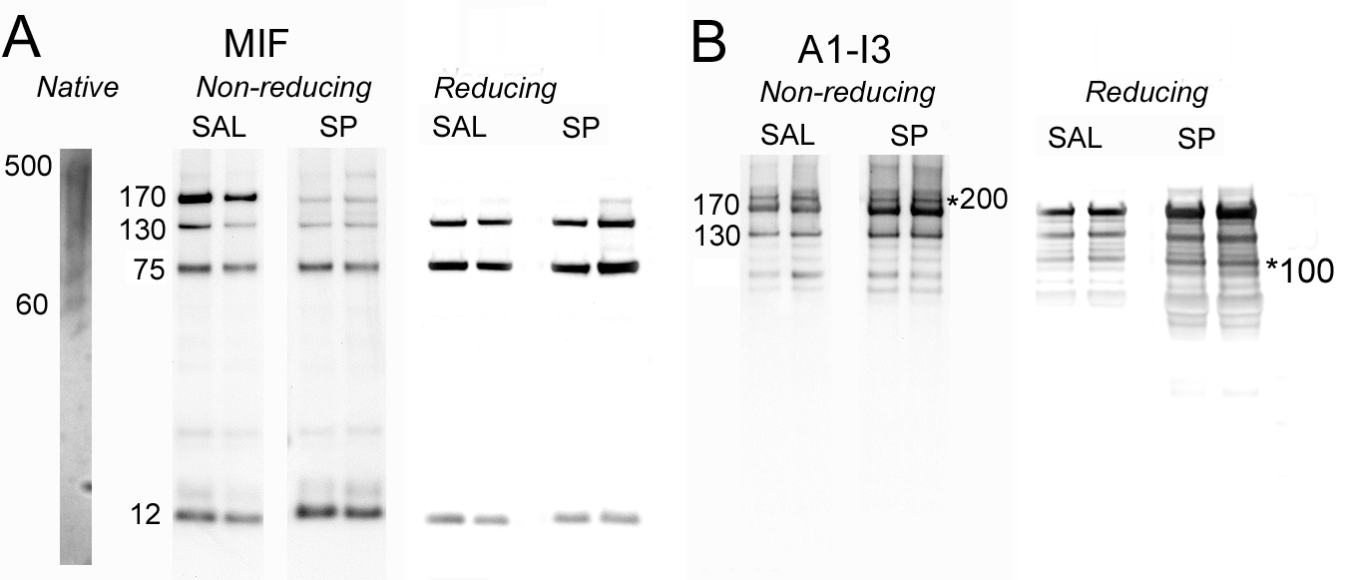
**MIF/A1-I3 complexes in bladder homogenates. **MIF WB under native, non-reducing and reducing conditions (A) showed that a broad band approximately 60–500 kDa under native conditions resolved into 4 distinct bands under non-reducing conditions. These bands had the apparent molecular weight of 170, 130, 75 and 12 kDa. The 12-kDa band corresponds well with monomeric MIF. Under reducing conditions, only two high-molecular weight MIF bands with apparent molecular weights of 130 and 75 kDa are present, in addition to the 12 kDa band. (B) A1-I3 WB under non-reducing conditions showed correspondence to the high-molecular weight MIF bands (170, 130 and 75) indicating MIF/A1-I3 complexes. In addition, a 200-kDa A1-I3 band (marked by asterisk) was not detected under MIF WB, indicating that this form of A1-I3 is not bound to MIF. Substance P treatment appeared to decrease the 170-kDa MIF band, while increasing the intensity of the 200-kDa and 170-kDa A1-I3 bands. Under reducing conditions, an A1-I3 band at 100 kDa (marked by asterisk) can also be observed. For non-reducing and reducing WB, representative WB from two different animals in each group are presented. Apparent molecular weights (in kDa) are indicated by numbers to the right of the lanes.

In whole bladder homogenates, MIF WB under native conditions revealed a broad band approximately 60–500 kDa (Fig 4A) indicating MIF formed large molecular weight complexes in the bladder, similar to our earlier observation in ILF [10]. Under non-reducing conditions, 4 prominent, distinct bands were observed corresponding to apparent molecular weights of 170, 130, 75 and 12-kDa (Fig. 4A). In addition, faint but distinct MIF bands were also localized at 24-kDa and 15-kDa.

A1-I3 WB revealed a banding pattern that was similar to that observed with MIF WB (Fig 4B) in that clear 75, 130 and 170-kDa bands were observed matching the MIF bands. However, a 200-kDa band (not matching MIF) was observed and there was a lack of a 12-kDa band (monomeric MIF), as expected. In fact, there were no bands below 60-kDa.

**Figure 5:**
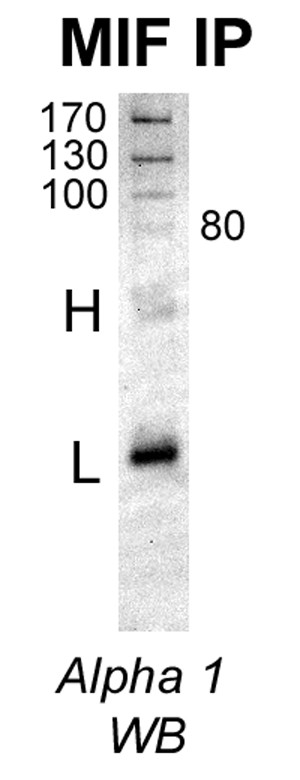
**Co-immunoprecipitation of MIF/A1-I3 from bladder homogenates. **Co-immunoprecipitation of bladder homogenates followed by A1-I3 WB showed that MIF antisera was able to pull-down MIF/A1-I3 complexes with apparent molecular weights of 170, 130, 100 and 80 kDa. H, L labels refer to heavy chain and light chain of IgG, respectively. A1-I3 WB was performed under reducing conditions.

Co-immunoprecipitation of whole bladder homogenates using MIF antisera followed by A1-I3 WB, showed 4 distinct bands of 170, 130, 100 and 75-kDa (Fig 5). Thus, we confirmed that 170,130 and 75-kDa bands are MIF/A1-I3 complexes. The appearance of an A1-I3 band at 100-kDa likely represents protease activation of A1-I3 [19].

### MIF complexes in serum, urine and intraluminal fluid

**Figure 6:**
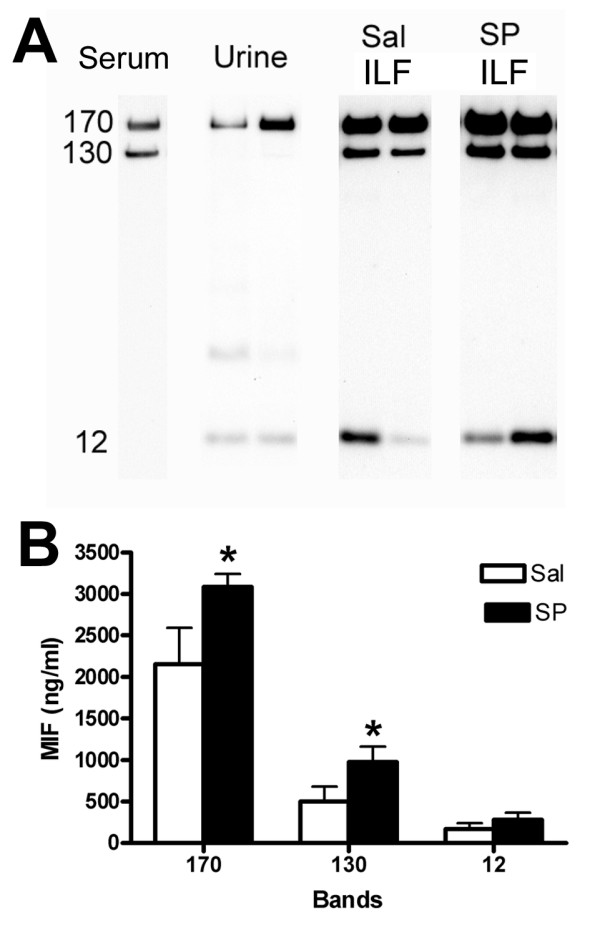
**Substance P-induced release of MIF/A1-I3 complexes in the intraluminal fluid. **A) MIF WB of serum, urine collected before treatment and intraluminal fluid of saline treated vs SP-treated rats demonstrated differences in the forms of MIF found. Serum only contained high-molecular weight MIF complexes of 170 and 130-kDa apparent molecular weight. No 12-kDa MIF was detected in the serum. In pre-treatment urine, however, only 170-kDa MIF/A1-I3 (as verified by A1-I3 WB; data not shown) and 12-kDa MIF bands but no 130-kDa were observed. B) In the intraluminal fluid (ILF), 170 and 130-kDa bands, corresponding to MIF/A1-I3 complexes [10] were noted along with 12-kDa MIF. SP treatment increased both 170 and 130 kDa MIF/A1-I3 amounts in the ILF (Figure B).

MIF non-reducing WB of serum, pre-treatment urine and ILF (in saline and SP-treated rats) showed a similar pattern to that reported by us previously [10], however, some differences were observed. Briefly, serum showed only 170 and 130-kDa MIF bands, but no 12-kDa MIF (Fig 6), while urine showed only 170 and 12-kDa MIF. The ILF, in agreement with our previous report [10] showed all three MIF bands. A MIF band at 75-kDa was not observed in any of the fluids examined, indicating that this complex is only present in the bladder. Previous mass-spectroscopy analyses demonstrated that 170 and 130 bands correspond to A1-I3 in the ILF [10], and A1-I3 WB showed corresponding bands in the present study for urine.

### MIF protein amounts in bladder and ILF as determined by ELISA or Western-blotting

**Figure 7:**
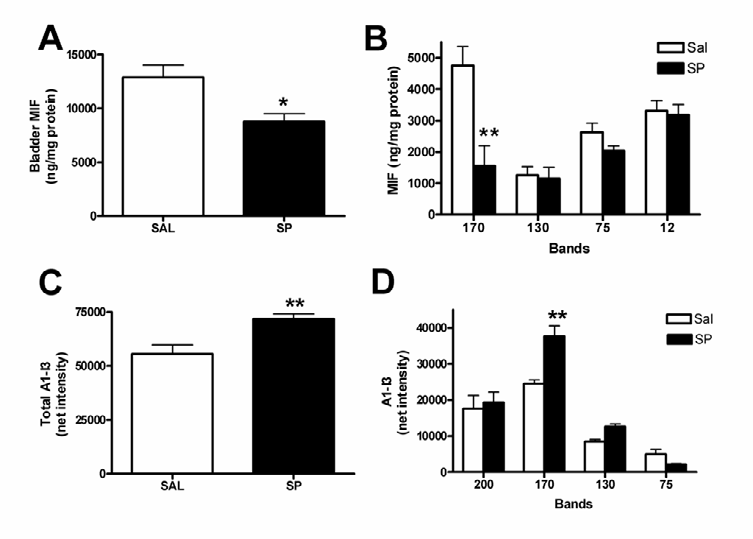
**Effect of Substance P-treatment on specific MIF/A1-I3 complexes. **Substance P treatment decreased the total MIF detected in bladder homogenates (using WB under non-reducing conditions) and this decrease was statistically significant (A). In addition, examining the changes in individual bands (under non-reducing conditions) showed that substance P treatment caused a significant decrease in the 170-kDa band in bladder homogenates, while changes in other bands did not reach statistical significance (B). SP treatment also increased the total amount of A1-I3 in the bladder, presumably due to plasma extravasation (C). In particular, the 170-kDa A1-I3 band was significantly increased (D). * = p < 0.05; ** = p < 0.01.

Both the ELISA and the WB findings showed decreased MIF in the whole bladder homogenates after SP while the amount in the ILF increased after SP (Table I; Fig 7A), in agreement with our previous reports [6,9,10]. However, the amounts reported by the ELISA were markedly less than the amount estimated by WB. The discrepancy was larger for the ILF, since the ELISA detected MIF protein levels that were 3 orders of magnitude less than those estimated by WB. There was no difference in the amount of MIF detected by ELISA (Table I) in intact bladders when compared to those treated with saline, indicating that our experimental manipulations (e.g. injections to withdraw urine and inject saline into the bladder), do not alter MIF levels.

Using densitometry analysis of the intensities of the different bands observed in WB we observed that in MIF WB, SP treatment markedly decreased the 170-kDa band intensity (Fig 7B) while changes in intensity in the other bands (130, 75 and 12-kDa) did not reach statistical significance. Similar analysis of the A1-I3 WB showed that SP treatment increased the total amount of A1-I3 in the bladder (Fig 7C) through a preferential increase in the intensity of the 170-kDA A1-I3 band while no statistically significant changes were detected in the other bands (Fig 7D).

## Discussion

In the present study, A1-I3 (a general protease inhibitor) was found in the lamina propria and interstitial spaces of intact bladders using immunohistochemistry. Since we documented that the bladder does not synthesize A1-I3, resident A1-I3 must be plasma derived, as has been also reported for the kidney [14]. In addition, double-immunofluorescence showed that, in intact and saline-treated rats, basal and intermediate cells in the urothelium contain only MIF (not A1-I3).

WB analysis under native conditions showed that there was no uncomplexed MIF (e.g. trimeric at 36 kDa, or monomeric at 12 kDa) in the bladder. Rather, MIF formed high-molecular weight complexes that under non-reducing conditions resolved into 12-kDa (monomeric) MIF as well as MIF/A1-I3 high-molecular weight bands indicating that MIF complexes are formed by noncovalent and covalent interactions (MIF/A1-I3 resistant to non-reducing western-blotting conditions). Co-immunoprecipitation with MIF antisera, followed by A1-I3 WB confirmed MIF/A1-I3 complexes in the bladder. Our results show that a considerable portion of the total MIF in the bladder is associated with A1-I3, even in intact and saline treated animals. Intact (not proteolytically activated) A1-I3 was present in whole bladder homogenates as having an apparent molecular weight of 200 kDa and did not have MIF attached to it. Lower molecular weight MIF/A1-I3 complexes (170, 130, and 75 kDa) were observed after non-reducing WB and likely reflect limited proteolysis of A1-I3 as described [19]. Therefore, A1-I3 activated by proteolytic activity is able to bind MIF in a process that occurs even in the absence of inflammation (intact bladder).

High molecular weight MIF complexes (170 and 130-kDa) and uncomplexed (12 kDa) MIF were also detected in the ILF, and we previously documented that these were MIF/A1-I3 complexes [10]. While serum also contained 170 and 130 kDa MIF, pre-treatment urine contained only 170 kDa MIF complex. Thus, we consider it likely that the 130 kDa MIF complex (which is increased during SP treatment) represents a fragment typically not released by the bladder, except after inflammatory stimuli (or experimental manipulation such as puncturing the bladder to empty of urine and refill with saline). The 75-kDa MIF/A1-I3 complex that is only found in the bladder and not in any of the fluids examined suggests that this fragment is an organ-specific proteolytic fragment that does not exit the bladder (presumably due to binding to membrane surfaces or intracellular location). Finally, 12-kDa MIF, while found in bladder homogenates, urine and ILF, was not detected in the serum by WB (non-reducing conditions) likely due to the detection limits of WB when compared to ELISA and/or the non-reducing conditions. Thus, similar to our recent findings in human serum [20], 12 kDa MIF represents only a small portion of the total MIF found in rat serum with MIF-protein complexes accounting for the majority of the MIF in serum. Since bladder epithelia *in vitro *[4] release 12-kDa MIF, we propose that MIF is similarly released from basal and intermediate cells in the rat urothelium (layers that show MIF immunostaining). However, it quickly forms non-covalent and covalent associations in the submucosa resulting in large-molecular weight complexes.

Treatment with SP increased MIF immunostaining in the lamina propria (also staining for A1-I3), and interestingly, MIF and A1-I3 co-localized in apical urothelial cells (cells normally devoid of either MIF or A1-I3). A1-I3 immunostaining was observed surrounding (but not intracellular, except for umbrella cells) urothelial cells after SP-treatment suggesting that A1-I3 and MIF/A1-I3 travel between basal and intermediate cells of the urothelium to reach the apical surface and/or the intraluminal space. A similar process of luminal entry of plasma proteins (particularly α2-macroglobulin) has been described for airway epithelia [21].

In agreement with our earlier findings, SP treatment decreased the amount of MIF in the bladder, as detected either by ELISA or WB (non-reducing conditions; Fig. 7A,B). However, a considerable discrepancy was noted between the ELISA MIF amounts and those estimated by WB (Table 1). We cannot fully account for this discrepancy, however, we also recently reported that a well-described ELISA to recognize human MIF in serum was able to detect less than 1% of the total MIF detected by WB [20]. Since the WB amounts in this study were estimated from a single point, it is possible that the estimates are not accurate when compared to the ELISA findings, although still reflecting changes within the WB (eg. saline vs SP). However, it is also likely that different antibodies (ELISA vs WB) may differ in their ability to recognize and bind to different forms of MIF (not just 12 kDa). Regardless of the explanation for the discrepancy, and even though the ELISA and WB findings agree in terms of the direction of the findings (i.e. SP decreased MIF amounts in the bladder), ELISA findings lack valuable information concerning changes in the different MIF complexes that are observed using WB (non-reducing).

**Figure 8:**
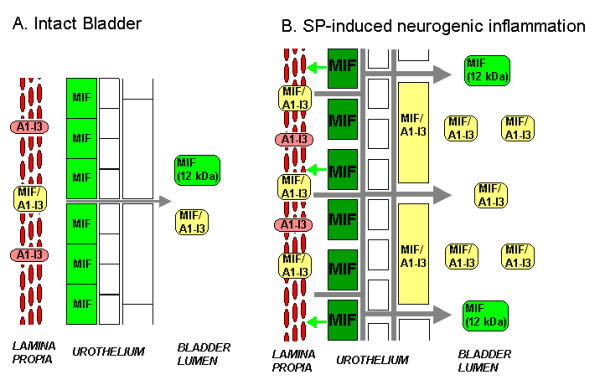
**Model of MIF/A1-I3 complex formation and paracellular transport through the urothelium. **In the intact bladder (A), A1-I3 (not synthesized by the bladder and most likely of liver origin) is located in the lamina propria immediately below the urothelium and throughout the interstitial spaces in the bladder. MIF is found in the urothelium, in the basal and intermediate cells. The lamina propia shows only weak MIF immunostaining. There is basal MIF release (and MIF/A1-I3 complexes) into the bladder lumen, however, A1-I3 is not located in the urothelium of intact rats. After SP-induced neurogenic inflammation (B), there is upregulation of MIF production in the urothelium, increased MIF staining in the lamina propria and greater release of MIF and MIF/A1-I3 complexes. Paracellular transport of A1-I3 can be observed in the urothelium. In addition, apical (umbrella cells) are now observed co-localizing both MIF and A1-I3.

SP upregulates MIF expression in the bladder [6], therefore, we propose (Fig. 8) that MIF produced in the bladder (most likely urothelial in origin) is released both into the lumen and into the lamina propria where it binds to proteolytically activated A1-I3 (resident in the interstitium, but of liver origin). During inflammation, while the total MIF content of the bladder decreases (a process that argues against entry of serum-derived MIF playing a significant role), the different MIF/A1-I3 complexes change differentially. SP-induced a decrease in resident 170-kDa MIF/A1-I3 complexes (likely the result of proteolytic processing), while increasing the luminal levels of 130-kDa MIF/A1-I3 and also 12-kDa MIF (non-covalently complexed MIF). MIF and MIF/A1-I3 complexes travel through the urothelium, enter the intraluminal space, and some of them localize to umbrella cells, previously devoid of MIF/A1-I3 (Fig. 8). Given that most of the MIF is found complexed to A1-I3, A1-I3 may thus serve as a mechanism to deliver MIF either to the intraluminal fluid or directly to umbrella cells.

Plasma extravasation in the bladder has been described by others [22], however, the process of MIF/A1-I3 travel through the urothelium described in this study involves exudation of protein complexes that are already resident in the intact bladder. We did observe an increase in the total amount of A1-I3 present in the bladder after SP treatment consistent with plasma extravasation, as would be expected. However, the total amount of MIF in the bladder (determined by ELISA and WB) decreased with SP treatment, which argues against plasma extravasation contributing to bladder MIF concentrations. Finally, the presence of specific MIF/A1-I3 complexes in the bladder only, when compared to serum or urine, suggest that processing of resident MIF/A1-I3 complexes (along with probable formation of new complexes during inflammation) account for the changes observed in this study.

The exact roles of the MIF/A1-I3 complexes or MIF non-covalently bound to other carrier protein in bladder inflammation remains to be determined. Our data in this study, along with our recent findings with experimental cystitis in rats [10] and bacterial cystitis in humans [8], suggest that rather than studying total MIF concentrations, particular attention needs to be paid to the associations of MIF with other proteins in order to increase our understanding of the *in vivo *processing and function of this cytokine. Transport of A1-I3 and/or MIF/A1-I3 complexes through the urothelium is a likely mechanism for the luminal entry of these complexes and their increase during inflammation. Such complexes appear to differentially bind to surface urothelial cells after SP-induced inflammation, either as a result of travel across the urothelium or from binding of soluble complexes in the intraluminal fluid. Our ELISA findings of decreased MIF in whole bladder homogenates confirm our previous reports [6,9,10] and suggest that while MIF and MIF/A1-I3 complexes are being excreted during SP-induced inflammation, the relative amount of MIF and/or MIF/A1-I3 binding to apical cells in the urothelium is probably small.

The receptor for MIF, CD74 has been recently identified [23] and we described increased expression of the receptor on urothelial cells after SP-induced inflammation [18]. Therefore, it is possible that MIF/A1-I3 binds to CD74 producing co-localization of MIF/A1-I3 in the apical urothelium after SP-induced inflammation. Alternatively, recent evidence showed that A1-I3 binds to cell-surface glucose regulated protein 78 (GRP78) receptors in prostate epithelia to activate signal transduction pathways [24]. GRP78 is an endoplasmic reticulum stress protein that is activated as part of the unfolded protein response in mammalian cells to prevent apoptosis [25-27]. However, GRP78 can also be localized to the surface of cells and be released into the circulation in inflammatory diseases [28-30]. The location of GRP78 has not been investigated in the bladder, but it is possible that SP-induced inflammation upregulates GRP78 in the urothelium thus accounting for MIF/A1-I3 binding to umbrella cells previously devoid of A1-I3. This possibility is currently being investigated and suggests that MIF and/or MIF/A1-I3 modulate inflammatory processes in the bladder through interaction with two different receptor systems, CD74 (recognized receptor for MIF, presumably able to bind MIF non-covalently bound to other carrier proteins) and/or GRP78 (recognized receptor for A1-I3 and presumably able to bind to MIF/A1-I3 complexes).

Regardless of the relative contribution of either receptor system to MIF-mediated bladder inflammation, sequestering intraluminal MIF with anti-MIF antibodies was able to reduce and/or reverse SP-induced inflammatory changes in the bladder [9]. However, determination of the contribution of either MIF acting through (presumably) CD74 receptors or MIF/A1-I3 complexes acting through (presumably) GRP78 receptors will help specify targets to reduce MIF-mediated inflammation. Recent evidence examining MIF complexes in human urine from UTI patients (e.g. MIF-ceruplasmin; MIF/α2-macroglobulin) suggest that interaction with other proteins may contribute to MIF's effect on this organ and may prove a general mechanism for MIF-mediated inflammation.

## Conclusion

A1-I3 (a general protease inhibitor of liver origin) and MIF/A1-I3 are resident in the interstitium, but not urothelium, in intact bladders. SP increased MIF immunostaining in the lamina propria, along with an increase in MIF/A1-I3 release into the intraluminal fluid, and co-localization of MIF/A1-I3 in umbrella cells previously devoid of either. Thus, A1-I3 may serve as carrier/delivery protein for MIF that is synthesized by urothelial cells.

## List of Abbreviations

A1-I3 = α1-I3

ABC = Avidin-biotinylated enzyme complex

ANOVA = Analysis of Variance

bp = base pair

CHAPS = 3-[(3-cholamidopropyl) dimethylammonio]-1-propane-sulfonate

ELISA = Enzyme linked immunosorbent assay

FITC = Fluorescein isothiocyanate

GRP78 = glucose regulated protein 78

i.p. = intraperitoneal

ILF = intraluminal fluid

kDa = kilodalton

LDL = low-density lipoprotein

LDS = lithium dodecyl sulfate

lp = lamina propria

MIF = Macrophage Migration Inhibitory Factor

PCR = Polymerase chain reaction

PVDF = Polyvinylidene fluoride

RNA = Ribonucleic acid

RT-PCR = Reverse transcriptase, polymerase chain reaction

s.c. = subcutaneous

SDS = sodium dodecyl sulfate

SEM = Standard error of the mean

SP = Substance P

TRITC = Tetramethylrhodamine isothiocyanate

WB = Western blotting

## Competing interests

The author(s) declare that they have no competing interests.

## Authors' contributions

PLV carried out the experimental inflammation experiments, conceived the study, performed the immunohistochemistry, participated in immunoblotting, performed the statistical analyses and drafted the manuscript. KLM participated in the inflammation model, performed ELISA, immunoblotting, RT-PCR, helped with the study design, analysis and helped draft the manuscript. Both authors read and approved the final manuscript.

## Pre-publication history

The pre-publication history for this paper can be accessed here:



## Figures and Tables

**Table 1:** Comparison of ELISA and Western blotting in determining MIF levels

	**Chemicon ELISA**	**Western Blotting**
**Bladder MIF (ng/mg protein)**		
**Saline (n = 5)**	694 ± 39.9	12870 ± 1150
**Substance P (n = 5)**	*398 *± 56.7**	*8765 *± 726.8*
**ILF MIF (ng/ml)**		
**Saline (n = 5)**	2.5 ± 0.8	2821 ± 635.2
**Substance P (n = 5)**	*19.0 *± 2.4***	*4338 *± 281.7*

* = p < 0.05; ** = p < 0.01; *** = p < 0.001
